# Supplementary material for: Phenotypic Analysis of Hematopoietic Stem and Progenitor Cell Populations in Acute Myeloid Leukemia Based on Spectral Flow Cytometry, a 20-Color Panel, and Unsupervised Learning Algorithms
Source: Int J Mol Sci. 2024 Feb 29;25(5):2847. doi: 10.3390/ijms25052847 (PMC10932439; doi:10.3390/ijms25052847)

Supplementary Material

Table S1: Patient characteristics

BM samples from 41 patients were used for this study and analyzed by the Routine Diagnostics laboratory. CD34pos HSPCs were determined (% of CD34pos HSPCs/Leukocytes) and percentages of CD19pos and myeloid HSPC subpopulations determined. In Case 6 (monoblastic AML) the leukemic blast population was CD34neg. MRD status was reporterd according to ELN guidelines.

The results of the MRD analysis with the 20-Color panel on the spectral cytometer are also given and discussed in the text of the Results section.

Table S1							
	Routine Diagnostics						20-color panel
		HSPCs %	Myeloid %	CD19pos %	MRD status by routine flow cytometry	Age	
1	AML, diagnosis	75.0	99.0	1.0		78	
2	AML, diagnosis	47.0	100.0	0.0		80	
3	AML, diagnosis	53.0	98.0	2.0		67	
4	AML, diagnosis	91.0	100.0	0.0		66	
5	AML, diagnosis	64.0	100.0	0.0		17	
6	AML, diagnosis	0.1	100.0	0.0		76	
7	AML, diagnosis	28.0	98.0	2.0		74	
8	AML, Post therapy	0.3	88.0	12.0	MRD neg	74	MRD neg
9	AML, Post therapy	0.2	94.0	6.0	MRD neg	64	MRD neg
10	AML, Post therapy	0.5	47.0	53.0	MRD neg	71	MRD neg
11	AML, Post therapy	0.5	83.0	17.0	MRD neg	46	MRD neg
12	AML, Post therapy	0.3	65.0	35.0	MRD neg	63	MRD neg
13	AML, Post therapy	1.6	27.0	73.0	MRD neg	49	MRD neg
14	AML, Post therapy	0.1	100.0	0.0	MRD neg	56	MRD neg
15	AML, Post therapy	0.3	95.0	5.0	MRD neg	70	MRD neg
16	AML, Post therapy	0.5	70.0	30.0	MRD neg	36	MRD neg
17	AML, Post therapy	0.5	53.0	47.0	MRD neg	20	MRD neg
18	AML, Post therapy	0.6	99.0	1.0	MRD neg	50	MRD neg
19	AML, Post therapy	1.0	78.0	22.0	MRD neg	55	MRD neg
20	AML, Post therapy	0.3	72.0	28.0	MRD neg	62	MRD neg
21	AML, Post therapy	0.2	60.0	40.0	MRD neg	71	MRD neg
22	AML, Post therapy	3.7	88.0	12.0	MRD neg	52	MRD neg
23	AML, Post therapy	0.5	100.0	0.0	MRD neg	69	MRD pos
24	AML, Post therapy	1.1	98.0	2.0	MRD pos	81	MRD pos
25	AML, Post therapy	6.0	98.0	2.0	MRD pos	74	MRD pos
26	AML, Post therapy	30.0	95.0	5.0	MRD pos	77	MRD pos
27	CMML	1.9	100.0	0.0		66	
28	MDS/MPS	0.4	100.0	0.0		74	
29	MDS/AML	6.9	100.0	0.0		75	
30	MGUS	nd				78	
31	Multiple Meyloma	nd				73	
32	Multiple Myeloma	nd				87	
33	isolated anemia	0.6	93.0	7.0		68	
34	LLA-T post therapy	1.1	34.0	66.0	MRD T neg	59	
35	LLA-T post therapy	1.3	80.0	20.0	MRD T neg	56	
36	LLA-B post therapy	10.4	24.0	76.0	MRD B neg	4	
37	LLA-B post-therapy	1.5	58.0	42.0	MRD B neg	67	
38	LLA-B post therapy	0.4	100.0	0.0	MRD B neg	23	
39	Isolated thrombopenia	0.5	100.0	0.0		90	
40	Post hepatic transplant	1.4	70.0	30.0		19	
41	Agranulocytosis	2.8	100.0	0.0		36	

Table S2: 10-color Antibody panels used for routine analysis
A: list of antibodies used in routine diagnostics
B: 10-color panels used in routine AML and MDS diagnostics. Panel 1 is used as a screening panel.

Table S2

A: Antibodies used for routine diagnosis

Antibody	Clone	Fluorochrome	Company
CD2	MT912	PE	Dialine
CD3	UCHT1	AA750	Immunotech
CD4	13B8.2	PC5.5	BC
CD7	8H8.1	APC700	BC
CD10	ALB1	APC700	BC
CD11b	BEAR1	FITC	BC
CD11c	S-HCL-3	PE	BD
CD13	SJ1D1	ECD	BC
CD14	RMO52	FITC	BC
CD15	80H5	PB	BC
CD16	3G8	APC750	BC
CD22	SJ10.1H11	APC700	BC
CD33	D3H260.251	PC5.5	Immunotech
CD34	581	PC7	BC
CD36	CB38	FITC	BD
CD42b	HIP1	FITC	BD
CD45	J33	KO	BC
CD56	N901	ECD	BC
CD61	VI-PL2	PE	Pharmingen
CD64	22	APC750	BC
CD71	YDJ1.2.2	APC750	BC
CD79a	HM47	APC	BC
CD91	A2MRd2	PE	BD
CD117	104D2D1	APC	BC
CD123	7G3	PC7	BD
CD200	MRC OX-104	PE	BD
CD300	UPH2	APC	eBiosciences
HLA-DR	Immu-357	PB	BC
TDT	HT-6	FITC	Dako
MPO	MPO-7	PE	Dako
Glycophorin	JC159	PE	Dako

B: Panels used for routine diagnosis

	FL-1	FL-2	FL-3	FL-4	FL-5	FL-6	FL-7	FL-8	FL-9	FL-10
Panel 1	CD14	CD19	CD13	CD33	CD34	CD117	CD7	CD16	HLA-DR	CD45
Panel 2	CD36	CD2	CD56	CD4	CD34	CD117	CD10	CD64	CD15	CD45
Panel 3	CD11b	CD200		CD33	CD34	CD117	CD19	CD3		CD45
Panel 4	CD14	CD91	CD56	CD33	CD34	CD300	CD16	CD64	HLA-DR	CD45
Panel 5	CD36	Glycophorin		CD33	CD34	CD117		CD71		CD45
Panel 6	CD42b	CD61	CD13	CD33	CD34	CD117		CD71		CD45
Panel 7	CD14	CD11c	CD56	CD33	CD123	CD300	CD16	CD64	HLA-DR	CD45
Panel 8 ic	TDT ic	MPO ic	CD3	CD33	CD34	CD79a ic	CD22	CD3 ic		CD45

Table S3: 20-color panel composition for analysis on the spectral cytometer.

Table S3

	Cat#	Fluorchrome	Marker	Clone	Manufacturer
0		**Brilliant Buffer**			
1	BL329210	BV421	CD200	OX-104	BD
2	R7-20003	cFluor V450	CD14	M5E2	Cytek
3	R7-20011	cFluor V547	CD45	HI30	Cytek
4	BL304136	BV650	CD45RA	HI100	BD
5	BL329223	BV711	CD64	10.1	BD
6	CR-20001	cFluor™ B515	CD3	SK7	Cytek
7	CR-20002	cFluor™ B532	CD15	W6D3	Cytek
8	CR-20003	cFluor™ BYG575	CD133	W6B3C1	Cytek
9	CR-20004	cFluor™ BYG610	CD117	104D2	Cytek
10	CR-20005	cFluor™ BYG667	CD56	5.1H11	Cytek
11	CR-20006	cFluor™ B690	HLA-DR	L243	Cytek
12	R7-20009	cFluor™ BYG710	CD19	HIB19	Cytek
13	CR-20007	cFluor BYG750	CD33	WM53	Cytek
14	CR-20008	cFluor BYG781	CD34	581	Cytek
15	CR-20009	cFluor™ R659	CD371	50C1	Cytek
16	R7-20029	cFluor™ R668	CD7	CD7-6B7	Cytek
17	CR-20010	cFluor™ R685	CD16	3G8	Cytek
18	R7-20013	cFluor™ R720	CD123	6H6	Cytek
19	CR-20011	cFluor™ R780	CD36	5-271	Cytek
20	CR-20012	cFluor™ R840	CD38	HB7	Cytek
21	R7-60008	ViaDye™ Red	DEAD		Cytek

Figure S1: Sequential gating strategy for CD34pos HSCP analysis

A: CD34pos HSPC are sequentially gated on TIME/SSC, SSC-H/SSC-A, FSC/SSC, CD34/SSC and backgated on CD45/SSC. The selected CD34pos HSPC population is then analyzed for the presence of myeloid (CD33pos), and lymphoid (CD19 pos), progenitors. In normal BM samples these two subpopulations show a ratio of approximately 2:1.

B: Analysis of a sample with an aberrant myeloid HSPC subpopulation. Analysis of the CD34pos HSPC population (violet) shows loss of the CD19pos subpopulation and presence only of myeloid HSPCs. These cells co-express CD117 and CD7. Whereas CD117 is physiologically co-expressed, CD7 constitutes an aberrant marker, since normal myeloid HSPCs do not express it.

Figure S1

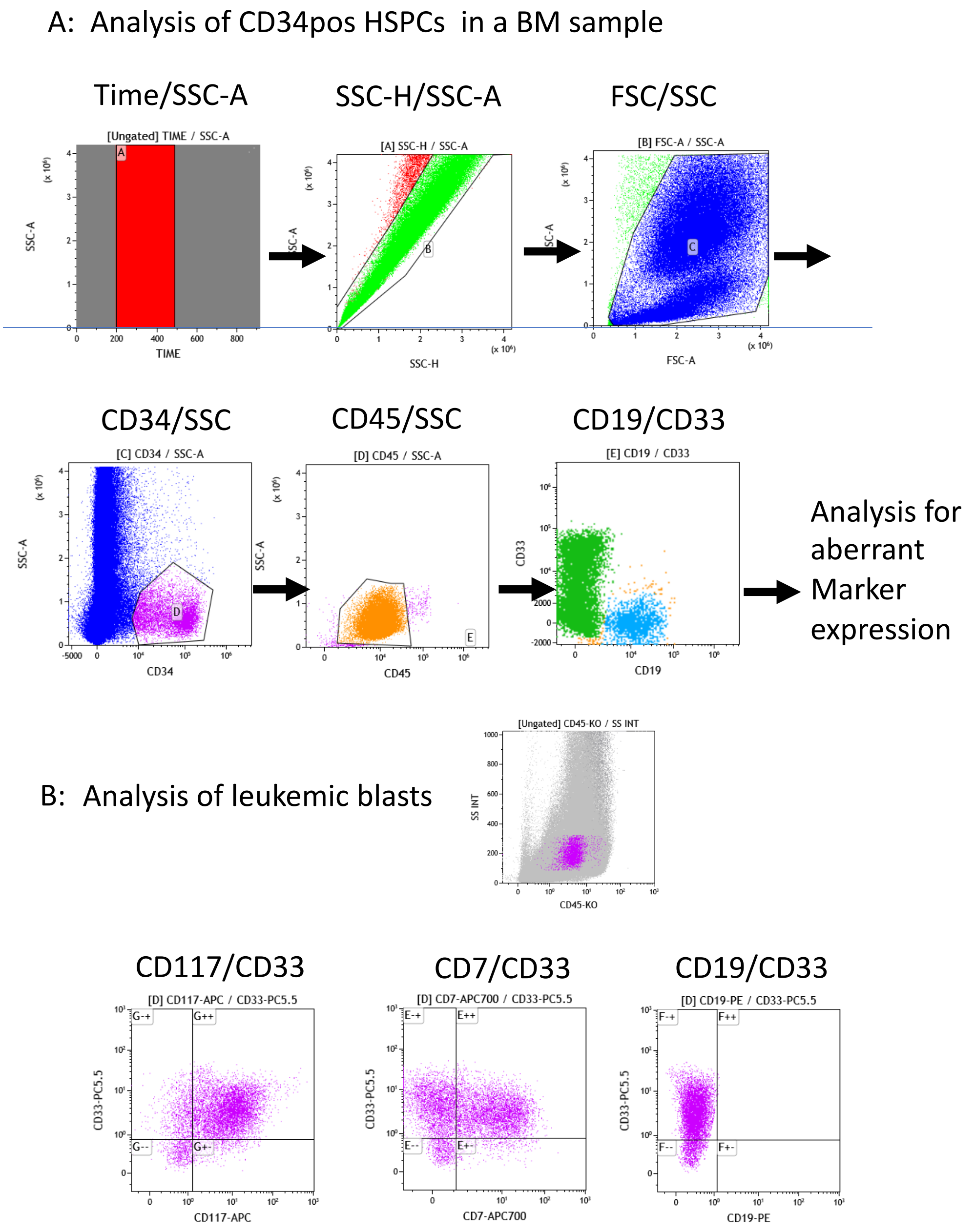

Figure S2: Analysis of CD123pos HSPCs.

Analysis of CD34pos HSPCs in CD371/CD123, CD38/CD123 and CD371/CD45RA 2D dotplots shows the CD123pos subpopulation (depicted in blue) in a characteristic position. HSC/MPP are depicted in brown.

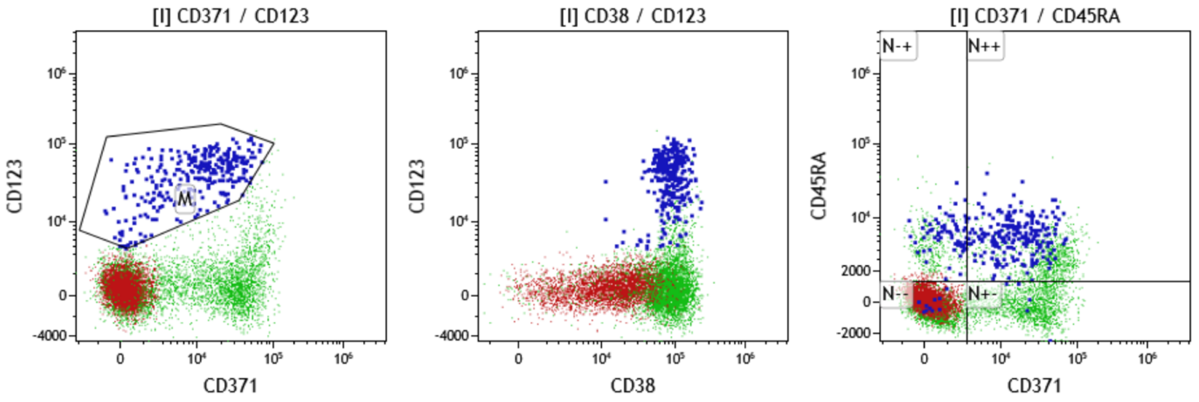

Figure S3: t-SNE CUDA analysis of normal BM samples.

Five normal BM samples were analyzed by t-SNE CUDA with $1x10^6$ cells used for the analysis (perplexity 30, iterations 1000). Results from a typical case are depicted.
Comparison of marker expression on the t-SNE plot with expression on 2D plots analyzed with KALUZA allowed the identification and annotation of 24 different populations in normal bone marrow samples. The different populations occupied exactly the same spot in the t-SNE plots from the five patients.

Figure S3

Identification of 24 different cell populations

1 Myeloid cells
2 HSPC
3 pDC
4 B lmphocytes
5 B lymphocytes
6 Myeloid cells CD200++
7 Myeloid cells
8 Myeloid precursors; CD16neg CD117pos
9 Myeloid cells
10 Dead cells + myeloid cells
11 T lymphopcytes
12 Eosinophils ?
13 Myeloid cells
14 Monocytes
15 Myeloid cells
16 Myeloid cells
17 NK cells
18 Myeloid cells CD16+++; apoptotic
19 B lymphocytes
20 Mast cells → 191 cells = 0.02%
21 Plasma cells
22 Basophils
23 Myeloid cells
24 Erythroid dells ?

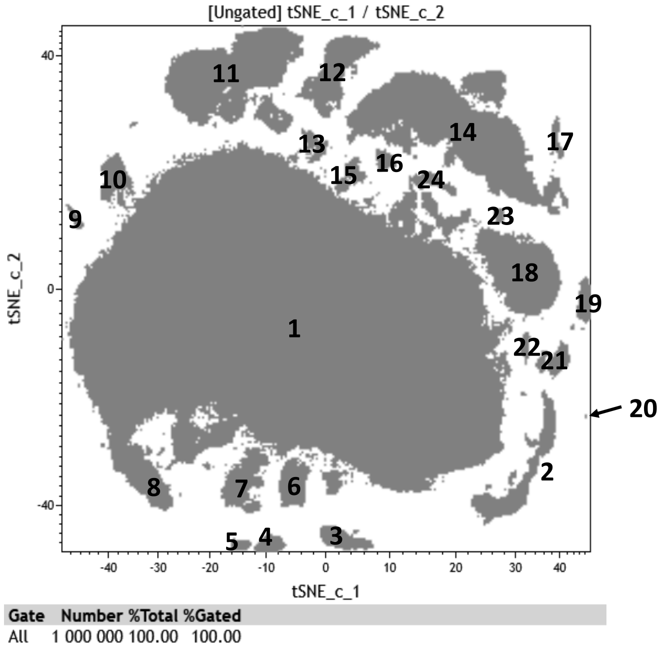

Figure S4: t-SNE CUDA analysis of CD34 HSPCs from normal bone marrow samples.

CD34pos HSPCs were gated according to Figure 1 from the five normal bone marrow samples and 4500 cells from each sample were used for t-SNE analysis (perplexity, 100 iterations 1000). Depicted are the t-SNE plots with all 20 parameters as z channel markers.

Figure S4

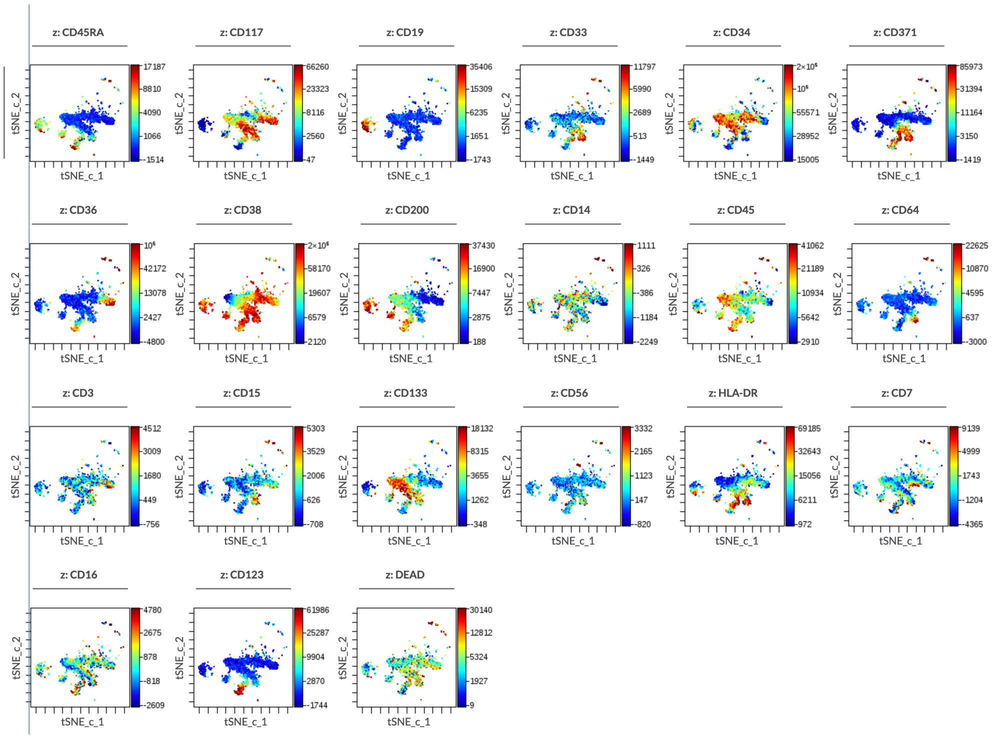

Supplement: Supplementary file 1 [file ijms-25-02847-s001.zip › ijms-2881771-supplementary.pdf]
